# Upregulation and differential expression of matrilysin (MMP-7) and metalloelastase (MMP-12) and their inhibitors TIMP-1 and TIMP-3 in Barrett's oesophageal adenocarcinoma

**DOI:** 10.1054/bjoc.2001.1929

**Published:** 2001-08

**Authors:** M T Salmela, M-L Karjalainen-Lindsberg, P Puolakkainen, U Saarialho-Kere

**Affiliations:** Departments of Dermatology, Helsinki, Finland; Departments of Pathology, Helsinki, Finland; Departments of Surgery, University of Helsinki, Helsinki, Finland; The Hope Heart Institute, Seattle, USA

**Keywords:** collagenase, in situ hybridization, laminin-5, tenascin-C

## Abstract

Oesophageal adenocarcinoma is believed to arise from metaplastic mucosa in the distal oesophagus, a condition also known as Barrett's oesophagus (BE). BE develops as a result of injury caused by refluxing gastric and duodenal contents and is associated with increased risk of malignant transformation. Matrix metalloproteinases (MMPs) have been implicated in all aspects of tumour progression; tumour growth, basement membrane degradation, invasion and metastatic spread. Using in situ hybridization, we investigated the expression patterns of collagenases-1 and -3, stromelysin-2, matrilysin, metalloelastase and TIMPs-1 and -3 in BE, adenocarcinoma and lymph-node metastases. Matrilysin was expressed abundantly in 12/15 tumours and in 4/6 lymph-node metastases and its expression correlated with the histological aggressiveness of tumour. Matrilysin and metalloelastase were upregulated already in BE. Stromelysin-2 and collagenase-3 expression was detected only in a few tumours. Collagenase-1 was expressed by cancer and stromal cells in 9/15 tumours. Tumour-infiltrating macrophages expressed metalloelastase in 13/15 cancers. TIMPs-1 and -3 were expressed in 12/15 and 11/15 tumours, respectively. Laminin-5 and tenascin were abundantly expressed at the invasive front of poorly differentiated tumours, but not in BE. Our results indicate that matrilysin is the principal MMP expressed by tumour cells in oesophageal adenocarcinoma, and further studies are needed to investigate whether matrilysin or tenascin-C could be used as a predictive marker for progression of BE to cancer. © 2001 Cancer Research Campaign http://www.bjcancer.com


*British Journal of Cancer* (2001) **85**(3), 383–392
© 2001 Cancer Research Campaign
doi: 10.1054/ bjoc.2001.1929, available online at http://www.idealibrary.com on IDEAL®

http://www.bjcancer.com

### Upregulation and differential expression of matrilysin (MMP-7) and metalloelastase (MMP-12) and their inhibitors TIMP-1 and TIMP-3 in Barrett's oesophageal adenocarcinoma

**MT Salmela[1], M-L Karjalainen-Lindsberg[2], P Puolakkainen[3,4] and U Saarialho-Kere[1]**

Departments of [1]Dermatology, [2]Pathology and [3]Surgery, University of Helsinki, Helsinki, Finland and [4]The Hope Heart Institute, Seattle, USA

**Summary** Oesophageal adenocarcinoma is believed to arise from metaplastic mucosa in the distal oesophagus, a condition also known as Barrett's oesophagus (BE). BE develops as a result of injury caused by refluxing gastric and duodenal contents and is associated with increased risk of malignant transformation. Matrix metalloproteinases (MMPs) have been implicated in all aspects of tumour progression; tumour growth, basement membrane degradation, invasion and metastatic spread. Using in situ hybridization, we investigated the expression patterns of collagenases-1 and -3, stromelysin-2, matrilysin, metalloelastase and TIMPs-1 and -3 in BE, adenocarcinoma and lymph-node metastases. Matrilysin was expressed abundantly in 12/15 tumours and in 4/6 lymph-node metastases and its expression correlated with the histological aggressiveness of tumour. Matrilysin and metalloelastase were upregulated already in BE. Stromelysin-2 and collagenase-3 expression was detected only in a few tumours. Collagenase-1 was expressed by cancer and stromal cells in 9/15 tumours. Tumour-infiltrating macrophages expressed metalloelastase in 13/15 cancers. TIMPs-1 and -3 were expressed in 12/15 and 11/15 tumours, respectively. Laminin-5 and tenascin were abundantly expressed at the invasive front of poorly differentiated tumours, but not in BE. Our results indicate that matrilysin is the principal MMP expressed by tumour cells in oesophageal adenocarcinoma, and further studies are needed to investigate whether matrilysin or tenascin-C could be used as a predictive marker for progression of BE to cancer. © 2001 Cancer Research Campaign http://www.bjcancer.com

**Keywords**: collagenase; in situ hybridization; laminin-5; tenascin-C

In contrast to the prevalence of oesophageal squamous cell carcinoma (SCC), that of oesophageal adenocarcinoma is rapidly increasing in Western countries. Oesophageal adenocarcinoma is thought to arise from metaplastic mucosa of intestinal-type located in the distal part of oesophagus above the lower oesophageal sphincter, a condition also known as Barrett's oesophagus (BE) (Jankowski et al, 1999). Replacement of normal squamous mucosa by Barrett's intestinal-type mucosa is believed to result from injury caused by gastric and duodenal contents regurgitating to oesophagus (Jankowski et al, 1999). The presence of specialized columnar epithelium of intestinal type is associated with increased risk of dysplastic changes and malignant transformation. Progression of BE to dysplasia and carcinoma is usually being followed by oesophagogastroscopies and biopsies but there are no reliable parameters to evaluate each patient's risk of developing oesophageal adenocarcinoma. The prognosis of Barrett's carcinoma is poor; 5-year survival is less than 15% (Antonioli and Wang, 1997), but if diagnosed and surgically treated early, Barrett's carcinoma is potentially curable (Peters et al, 1994). Despite intensive research, molecular events which take place during progression of normal squamous mucosa to Barrett's oesophagus and further to dysplasia and adenocarcinoma are not fully understood.

*Received 27 November 2000*
*Revised 28 March 2001*
*Accepted 5 April 2001*

*Correspondence to:* U Saarialho-Kere

Matrix metalloproteinases (MMPs) are a group of $Ca^{2+}$-dependent, zinc-containing enzymes which are altogether able to degrade all components of extracellular matrix (ECM) (Nagase and Woessner, 1999). MMPs participate in physiological processes like tissue morphogenesis, wound repair, and angiogenesis as well as in pathologic conditions such as arthritis, inflammatory bowel disease, atherosclerosis, and tumour growth and metastasis (Kahari and Saarialho-Kere, 1999). MMPs have been implicated in all aspects of tumour progression; they enhance tumour-induced angiogenesis, destroy local tissue architecture to allow tumour growth, and break down basement membranes in the process of metastatic spread. Different MMPs are often co-expressed in cancers in a cell-type specific manner and, thus, complement the proteolytic capacity of each other. The MMP family currently comprises 21 structurally related members which can be divided into collagenases (MMP-1, -8 and -13), gelatinases (MMP-2 and -9), stromelysins (MMP-3, -7, -10, -11, -12), membrane-type MMPs (MMP-14, -15, -16 and -17) and other MMPs (MMP-19, -20, -23 and -26) according to their structure and substrate specificity (Nagase and Woessner, 1999; Velasco et al, 1999; Park et al, 2000). MMPs not only degrade ECM components but they also modulate cell migration (Koshikawa et al, 2000) as well as immunologic response e.g. by shedding FasL (Tanaka et al, 1998) and they activate certain cytokines such as TNF-$\alpha$ and IGF (Chambers and Matrisian, 1997).

Collagenases are the principal proteinases that degrade native fibrillar collagens. Substrates of collagenase-1 include collagens I, II, III, VII, VIII and X, gelatin, aggrecan, versican, entactin, tenascin and proteoglycan link protein (Kahari and Saarialho-Kere, 1999). Collagenase-1 has been associated with poor prognosis in oesophageal cancers (Murray et al, 1998). Collagenase-3 has a broader substrate specificity being capable of degrading collagens I, II, III, IV, IX, X and XIV, gelatin, tenascin, fibronectin and laminin-1 (Kahari and Saarialho-Kere, 1999).

Stromelysin-2 (MMP-10) degrades in vitro e.g. collagen III and IV, gelatin, aggrecan, elastin, and proteoglycan core proteins (Murphy et al, 1991; Chandler et al, 1996). Expression of stromelysin-2 has been associated with invasion of epithelial cancers (Kerkela et al, 2001). Matrilysin is the smallest MMP and its in vitro substrates include proteoglycans, aggrecan, gelatin, fibronectin, tenascin and elastin as well as BM components nidogen, laminin and type IV collagen (Murphy et al, 1991). Matrilysin expression has been associated with epithelial tumours of the colon, prostate and breast (Fingleton et al, 1999). Furthermore, transfection of matrilysin cDNA enhances the invasive potential of colon cancer cells (Adachi et al, 1999). Human macrophage metalloelastase (MMP-12) is the most elastolytic MMP, but it also degrades type IV collagen, laminin-1, fibronectin, vitronectin, proteglycans and, like matrilysin, it cleaves plasmin to angiostatin (Chandler et al, 1996; Gronski et al, 1997).

The activity of MMPs is regulated at the transcriptional level by cytokines and growth factors and after secretion by proenzyme activation or by their natural tissue inhibitors, TIMPs-1, -2, -3 and -4, which inhibit MMPs by binding covalently to the active site of the enzyme. TIMP-1 is a very potent inhibitor of MMPs except MT-MMPs. TIMP-3 inhibits the activity of MMPs-1, -2, -3, -9 and -13 (Apte et al, 1996) but also the activity of MT-MMPs as well as TNF-$\alpha$ converting enzyme (TACE). However, the role of TIMPs is not restricted to the inhibition of MMPs. They possess growth promoting activities for various cell types as well and have antiangiogenic properties and promote apoptosis (Martin et al, 1996; Anand-Apte et al, 1997; Ahonen et al, 1998).

In this work the expression patterns of collagenase-1 (MMP-1) and -3 (MMP-13), stromelysin-2 (MMP-10), metalloelastase (MMP-12), matrilysin (MMP-7) and their tissue inhibitors, TIMP-1 and -3, were investigated in Barrett's oesophagus and adenocarcinoma. Furthermore, since the expression of tenascin-C and laminin-5 in tumors has been shown to correlate with high risk of invasiveness in certain cancer types (Pyke et al, 1995; Jahkola et al, 1996), their expression was also investigated. Our results demonstrate that matrilysin and metalloelastase are already upregulated in intestinal metaplasia and that TIMPs-1 and -3 are differentially regulated during transformation.

### MATERIALS AND METHODS

### Tissue samples

Patients were followed for Barrett's intestinal metaplasia, diagnosed by an experienced pathologist, or surgically treated for Barrett's oesophageal adenocarcinoma in Helsinki University Central Hospital, Finland. Formalin-fixed, paraffin-embedded specimens of Barrett's oesophageal adenocarcinoma ($n$ = 16, 6 with lymph node metastasis) and intestinal metaplasia ($n$ = 5) were obtained from the Department of Pathology, University of Helsinki. 8 of the samples were biopsies taken during upper gastrointestinal endoscopy and 13 were surgical specimens. In situ hybridizations for matrilysin and metalloelastase were performed also on additional biopsy specimens ($n$ = 10) taken during follow-up of 10 patients, which later developed matrilysin-positive Barrett's carcinoma. 4 samples only contained intestinal metaplasia, while 6 of them were follow-up biopsies in which the later operated cancer was initially diagnosed. 6 samples of normal oesophageal mucosa ($n$ = 6) were also examined.

Definition used for BE was presence of specialized intestinal-type columnar epithelium with mixed population of goblet, Paneth and endocrine cells lining a segment of distal oesophagus above the lower oesophageal sphincter. TNM-classification for each tumour was based on findings during operation and on pathologist's analysis. Preoperative CT-scan and abdominal ultrasound investigations were performed in all cases.

### In situ hybridization

The production and specificity of the anti-sense collagenase-1, collagenase-3, metalloelastase, matrilysin, stromelysin-2, as well as TIMP-1 and -3 probes have previously been described (Saarialho-Kere et al, 1994, 1996; Vaalamo et al, 1996, 1997, 1998; Airola et al, 1998). In situ hybridization was performed on 4-μm sections. Following deparaffinization and rehydration all samples were treated with proteinase K and were washed in 0.1 mol $l^{-1}$ triethanolamine buffer containing 0.25% acetic anhydride. Sections were hybridized overnight at 50˚C to 55˚C with $^{35}$S-labelled RNA probe. After hybridization, slides were washed under stringent conditions and treated with RNAse A to remove unhybridized probe. After 20 to 40 days autoradiographic exposure, the photographic emulsion was developed and the slides were stained with haematoxylin and eosin. Previously positive samples for each anti-sense probe were used as positive controls (colon cancers for TIMP-3, sweat gland tumours for matrilysin, chronic wounds for TIMP-1, collagenase-1 and stromelysin-2, squamous cell carcinomas for collagenase-3, and sarcoid granulomas for metalloelastase). The slides were independently assessed by two experienced investigators (U S-K, M-L K-L).

### Immunohistochemistry

Macrophages were stained using a monoclonal antibody (KP-1, Dako Corp, Carpinteria, CA, product M814), which reacts with CD68, a specific macrophage marker. The cells producing laminin-5 were detected with polyclonal antibodies against the $\gamma$-2 chain of laminin-5, provided by Prof Karl Tryggvason, Karolinska Institut, Stockholm, Sweden (Pyke et al, 1994). The extracellular matrix component tenascin-C was stained by using mouse monoclonal antibodies (no. 1927, Chemicon, Temecula, CA).

Anti-CD68 antibody was diluted 1:400, laminin-5 1:500 and tenascin 1:2000. CD68 and tenascin antibodies reacted for 1 h at 37˚C and antibody for laminin-5 reacted overnight at 4˚C. Immunohistochemistry was performed using the avidin–biotin–peroxidase complex technique (Saarialho-Kere et al, 1992). Sections were pretreated with 10 mg $ml^{-1}$ trypsin. Diaminobenzidine (DAB) was used as chromogenic substrate and sections were counterstained with Harris haematoxylin. Controls were performed with rabbit pre-immune serum or normal mouse immunoglobulins.

 © 2001 Cancer Research Campaign

**Table 1** TNM classification and tumour grading for each sample

| Gender | Age | TNM | Survival | Differentiation | MMP-1 | MMP-13 | MMP-7 | MMP-10 | MMP-12 | TIMP-1 | TIMP-3 | Tenascin | Laminin-5 |
|---|---|---|---|---|---|---|---|---|---|---|---|---|---|
| F | 67 | IM, no dysplasia | | | N.D. | – | N.D. | – | N.D. | – | + | – | – |
| M | 50 | IM, no dysplasia | | | + | – | + | – | + | + | ++ | – | – |
| F | 53 | IM, no dysplasia | | | – | – | + | – | (+) | + | + | – | – |
| M | 68 | IM, no dysplasia | | | – | – | – | (+) | ++ | – | + | – | – |
| M | 70 | IM, no dysplasia | | | – | – | – | – | – | – | – | – | – |
| M | 48 | IM, mild dysplasia | | | – | (+) | – | – | + | (+) | ++ | – | – |
| M | 73 | T3N1M0 with M | N.D. | poor | ++ | + | +++ | – | + | + | + | + | +++ |
| M | 52 | T3N0M0 | 27 † | poor | ++ | – | +++ | – | ++ | +++ | – | (+) | + |
| M | 57 | T3N1M0 | 4 † | poor | – | – | +++ | N.D. | +++ | – | +++ | +++ | ++ |
| | | T3N1M0 with M | 4 † | poor | + | + | + | – | – | + | – | + | ++ |
| | | T3N1M0 | 4 † | high | + | + | + | – | ++ | – | +++ | (+) | (+) |
| M | 53 | T3N1M0 with M | 24 | poor/high | + | + | +++ | + | +++ | ++ | ++ | +++ | ++ |
| | | T3N1M0 with M | 24 | poor/high | + | – | +++ | + | ++ | (+) | ++ | +++ | ++ |
| M | 63 | T3N1M0 with M | 18 | poor/high | +++ | (+) | + | + | +++ | ++ | +++ | + | + |
| M | 69 | T2N0M0 | 24 | high | – | – | + | – | + | ++ | ++ | (+) | – |
| M | 78 | T2N1M0 | 30 † | high | – | – | – | – | – | – | – | – | – |
| M | 63 | T2N0M0 | 16 | high | – | – | + | – | + | – | + | + | – |
| M | 52 | T4N0M0 | 37 | high | (+) | – | ++ | – | ++ | ++ | ++ | + | ++ |
| M | 77 | T3N1M1 | N.D. | high | + | – | + | – | – | ++ | +++ | (+) | – |
| | | T3N1M1 with M | N.D. | high | + | – | – | – | (+) | + | ++ | – | (+) |
| M | 64 | T2N0M0 | 2 † | high | ++ | – | +++ | – | + | ++ | ++ | + | ++ |
| M | 70 | T1N1M0 | 23 | high | + | + | + | – | – | (+) | ++ | + | (+) |
| M | 50 | T3N1M0 with M | 5 | high | – | – | ++ | – | ++ | ++ | ++ | ++ | ++ |
| M | 81 | TxNxM0 | 14 | high | – | – | – | – | + | – | – | + | – |
| F | 82 | TxN0M0 | 24 | high | – | – | – | + | + | + | – | (+) | – |
| Tumours positive for each probe or antibody used | | | | | 9/15 | 4/15 | 12/15 | 3/15 | 13/15 | 12/15 | 11/15 | 14/15 | 10/15 |

N.D., not determined; –, no signal detected; (+), signal in occasional cells; +, specific signal in low number of cells; ++, specific signal in moderate number of cells; +++, specific signal in high number of cells; IM, intestinal metaplasia; M, metastasis. Survival time is stated in months.

© 2001 Cancer Research Campaign

### RESULTS

### Matrilysin is expressed by malignant cells in Barrett's carcinoma and is up-regulated early in oncogenesis

Matrilysin mRNA was detected in 12/15 samples of Barrett's carcinoma. Most abundant expression was detected in poorly differentiated tumours (Table 1; Figure 1A). Matrilysin was expressed throughout the tumour, not only at the invasive border (Figure 1A).

mRNA for matrilysin was seen already in samples of intestinal metaplasia (Table 1; Figure 2A,B) suggesting that it is upregulated early in oncogenesis. Furthermore, in situ hybridization on follow-up biopsies showed matrilysin expression in 3/6 biopsies with adenocarcinoma and in 2/4 biopsies with intestinal metaplasia (data not shown). All 10 patients followed by biopsies later developed matrilysin-positive adenocarcinoma of oesophagus.

Positive signal for matrilysin was seen in 4/6 lymph nodes with metastatic tissue (Figure 2C). Primary tumours from which metastases originated were also highly positive for matrilysin mRNA. No signal for matrilysin mRNA was detected in normal oesophagus. Tenascin-C protein was produced bordering or inside areas abundant with matrilysin mRNA positive cells (Figures 1A, F, 2D, E).

### Collagenase-1 is expressed by malignant cells, but stromelysin-2 and collagenase-3 are rarely detected in Barrett's carcinoma

Collagenase-1 mRNA was seen in 9/15 tumours. Carcinoma cells (Figure 2F,G), as well as stromal fibroblast-like cells (data not shown) expressed collagenase-1. No signal for collagenase-1 mRNA was seen in lymph node metastases or in samples with plain intestinal metaplasia (Table 1).

Signal for stromelysin-2 and collagenase-3 were detected only in 3/15 and 4/15 tumours, respectively, and never in metastases (Table 1). Expression of stromelysin-2 was seen in luminal surface area of tumours, not in the invasive border (data not shown). Collagenase-3 was detected in occasional fibroblast-like cells of the stroma (data not shown). Signal for stromelysin-2 or collagenase-3 mRNAs was not detected in intestinal metaplasia (Table 1).

### Metalloelastase is expressed by a subset of macrophages

MMP-12 mRNA was detected in 13/15 tumours. Particularly, well-differentiated tumour regions had lots of infiltrating MMP-12 -positive cells (Figure 1B; 3A,B). As assessed with immunostaining for CD68 (Figure 3C,D), MMP-12 was produced by a subset of macrophages. Signal for MMP-12 mRNA was seen also in 3/6 lymph node metastases (Figure 3E). MMP-12 expression was detected also in 4/6 samples of intestinal metaplasia (Figure 3F,G) but the number of positive cells was greater in cancer (Table 1). Follow-up biopsies showed expression of MMP-12 in 3/6 biopsies with adenocarcinoma and in 2/4 biopsies with intestinal metaplasia (data not shown).

### TIMP-1 and -3 are expressed by fibroblast-like cells within the tumour

TIMP-1 expression was detected in 12/15 tumours (Figures 1C, 4A). It was not expressed in the surface of tumours, but abundantly in deeper areas of the mucosa, particularly, in aggressive tumours with poor histological differentiation (Figure 1C). TIMP-1 was detected in stromal cells surrounding glandular structures in 4/6 lymph-node metastases (Figure 4H). Occasional signal for TIMP-1 was observed in stromal cells between glands in 3/6 intestinal metaplasia samples, in which the degree of expression was very low compared to malignant samples (data not shown, Table 1).

Signal for TIMP-3 mRNA was detected in 11/15 tumours (Figures 1D, 4C). Cells expressing TIMP-3 were stromal, fibroblast-like cells (Figure 4D). In poorly differentiated tumours, TIMP-3 was expressed by stromal cells throughout the malignant tissue, whereas in tumours with high differentiation, cells positive for TIMP-3 mRNA lined the malignant cell islands (Figure 1D). Expression of TIMP-3 was detected in 5/6 samples of intestinal metaplasia (Figure 4F, G) and in 5/6 lymph node metastases (Figure 4I).

### Protein expression of tenascin-C and laminin-5 is most prominent in poorly differentiated tumours

Tenascin-C immunoreactivity was detected in 14/15 tumours, but no staining was seen in samples of intestinal metaplasia. Expression was abundant particularly in poorly differentiated tumours (Figure 1F), where it seemed to form net-like structures between invasive cells, which were positive for laminin-5 and matrilysin mRNA (Figure 1A, E).

Positive laminin-5 staining was detected in tumour cells particularly at the invasive front (Figure 1E). Matrilysin mRNA and laminin-5 protein partly colocalized (Figure 1A, E). No staining for laminin-5 was seen in intestinal metaplasia (data not shown).

### DISCUSSION

Compared to the normal population, patients with Barrett's oesophagus have up to 125-fold increased risk for developing oesophageal adenocarcinoma (Bonelli 1993). Patients followed endoscopically have better prognosis than other patients who develop oesophageal adenocarcinoma (Peters et al, 1994; van Sandick et al, 1998). During endoscopic examination several biopsies are taken, but the risk of missing the exact dysplastic or malignant tissue is still present. Furthermore, lack of reliable parameters to evaluate each BE patient's risk of developing invasive tumour, has encouraged to search new markers to help the evaluation.

In situ hybridization is a useful method for studying expression of MMPs, because transcriptional regulation is an important pathway for MMP induction (Westermarck and Kahari, 1999). Instead of storing enzymes in cytoplasmic structures, MMPs and TIMPs studied in this work are secreted readily to the extracellular space. Only one previous study on MMPs in oesophageal adenocarcinoma exists. By immunohistochemistry Murray et al (1998) found that MMP-1, MMP-2 and MMP-9 are expressed in 30%, 81% and 78% of oesophageal adenocarcinomas, respectively. They also found that expression of MMP-1 correlates with poor prognosis in oesophageal cancers. Furthermore, Shima et al (1992) found that in oesophageal SCC, expression of 72-kDa gelatinase (MMP-2) and stromelysin-1 (MMP-3) is associated with lymph-node metastasis and vascular invasion.

Matrilysin was abundantly expressed in our material, particularly in poorly differentiated tumours which had strong immunostaining for laminin-5 at the invasive border. Matrilysin

 *© 2001 Cancer Research Campaign*

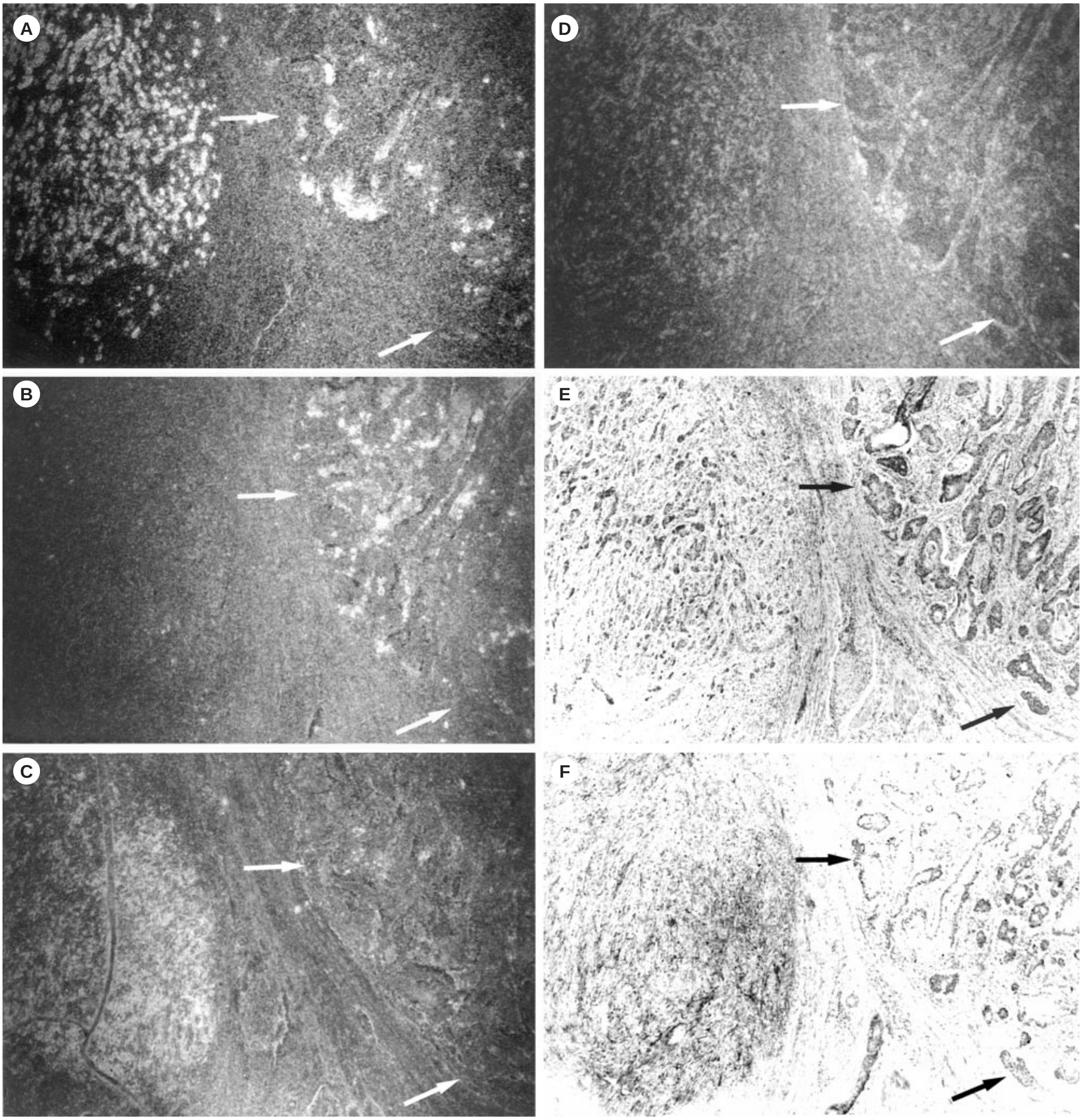

**Figure 1** Expression patterns of MMPs-7 and -12, TIMPs-1 and -3 and laminin-5 as well as tenascin in Barrett's cancer. (**A**) Matrilysin expression by carcinoma cells in a poorly differentiated tumour. Well-differentiated area on the right side shows much lower matrilysin expression. (**B**) MMP-12 mRNA is produced by stromal cells surrounding neoplastic glands in the well-differentiated part of the same tumour. In the aggressive area of tumour, MMP-12 expression is nearly absent. (**C**) TIMP-1 is expressed particularly in the poorly differentiated area. (**D**) Expression of TIMP-3 is abundant in poorly differentiated as well as well-differentiated areas. (**E**) Laminin-5 is produced by invading tumour cells implicating their migratory status. Production of laminin-5 partly colocalizes with that of matrilysin (**A**). (**F**) Tenascin-C is produced at the poorly differentiated area of the tumour particularly at the invasive border. Arrows depict corresponding spots. Magnification x20 (**A–F**)

has recently been reported at protein level in oesophageal SCC, in which its expression correlates with poor prognosis (Yamamoto et al, 1999). While this study was in progress Yamashita et al (2000) showed by Northern hybridization and Ohashi et al (2000) by immunohistochemistry that matrilysin expression status might be a prognostic factor in patients with oesophageal squamous cell carcinoma. Laminin-5 is often considered as a marker for invasiveness (Skyldberg et al, 1991; Pyke et al, 1995) since invading malignant cells express and adhere to laminin-5 (Pyke et al, 1994) and migrate on it (Tani et al, 1997). The role of matrilysin in Barrett's carcinoma is most likely the same as in gastric and colon cancers. Matrilysin increases the invasive potential of malignant colon cancer cells (Adachi et al, 1999), but is also required for tumour initiation and growth (Fingleton et al, 1999). In addition to its degradative effects on ECM, matrilysin also activates proMMP-2, which is known to be produced in 81% of oesophageal adenocarcinomas (Murray et al, 1998).

*© 2001 Cancer Research Campaign*

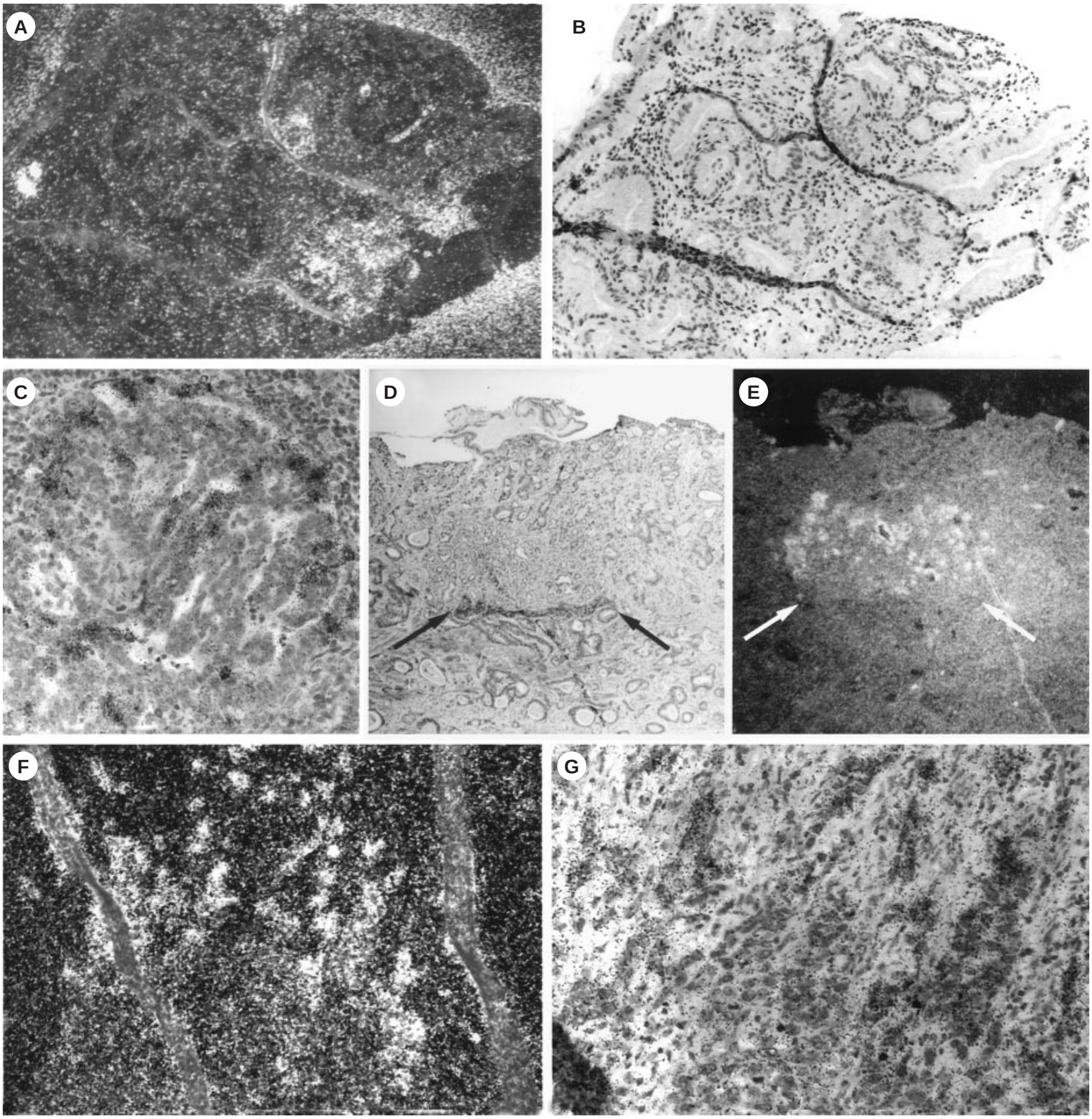

**Figure 2** Matrilysin and collagenase-1 expression in Barrett's carcinoma. (**A**) Matrilysin expression in intestinal metaplasia by epithelial cells. (**B**) Corresponding bright-field image. (**C**) Bright-field image of a lymph node metastasis with matrilysin expressing cells. (**D**) Tenascin-C positive area is in immediate contact with matrilysin expressing tumour cells. (**E**) Expression of matrilysin in another poorly differentiated tumour. (**F**) Collagenase-1 expression in a poorly differentiated tumour in the invading carcinoma cells. (**G**) Corresponding higher magnification bright-field image. Arrows depict corresponding spots (**D, E**). Magnification x20 (**D, E**), x40 (**A, B**) x100 (**F**) and x200 (**C, G**)

As in preinvasive lesions of colorectal carcinoma, adenoma and carcinoma in situ (Fingleton et al, 1999), we found matrilysin expression in premalignant lesions of the oesophagus. This suggests that metaplasia-dysplasia-adenocarcinoma sequence in oesophagus has some features in common with colorectal adenoma-carcinoma sequence and that matrilysin might be significant also in development of BE and oesophageal adenocarcinoma. Another feature in common with colorectal cancer is APC-gene mutations (Mueller et al, 2000), which lead to accumulation of beta-catenin. Together with the DNA-binding protein TCF-4, β-catenin functions as a transcriptional activator for matrilysin (Brabletz et al, 1999).

Tenascin-C is a large glycoprotein of the extracellular matrix with growth-promoting and anti-adhesive functions. Tenascin-C has previously been demonstrated to be involved in carcinogenesis in other types of cancer (Jahkola et al, 1996). Interestingly, our novel results show that tenascin-C is produced particularly in aggressive and poorly differentiated oesophageal adenocarcinomas, but not in intestinal metaplasia. Cells in these areas also stained with laminin-5, which implicates migrating status of malignant cells in contact with tenascin-C protein. Tenascin-C may help invading cells detach from surrounding matrix and cells. Of the MMPs, only matrilysin was systematically expressed in the same regions as tenascin-C. Matrilysin is known to degrade both large and small tenascin-C isoforms, and large isoform is cleaved also by MMP-2 and MMP-3 (Siri et al, 1995). Tenascin is also degraded by MMP-1 and MMP-13, but neither of them was

 *© 2001 Cancer Research Campaign*

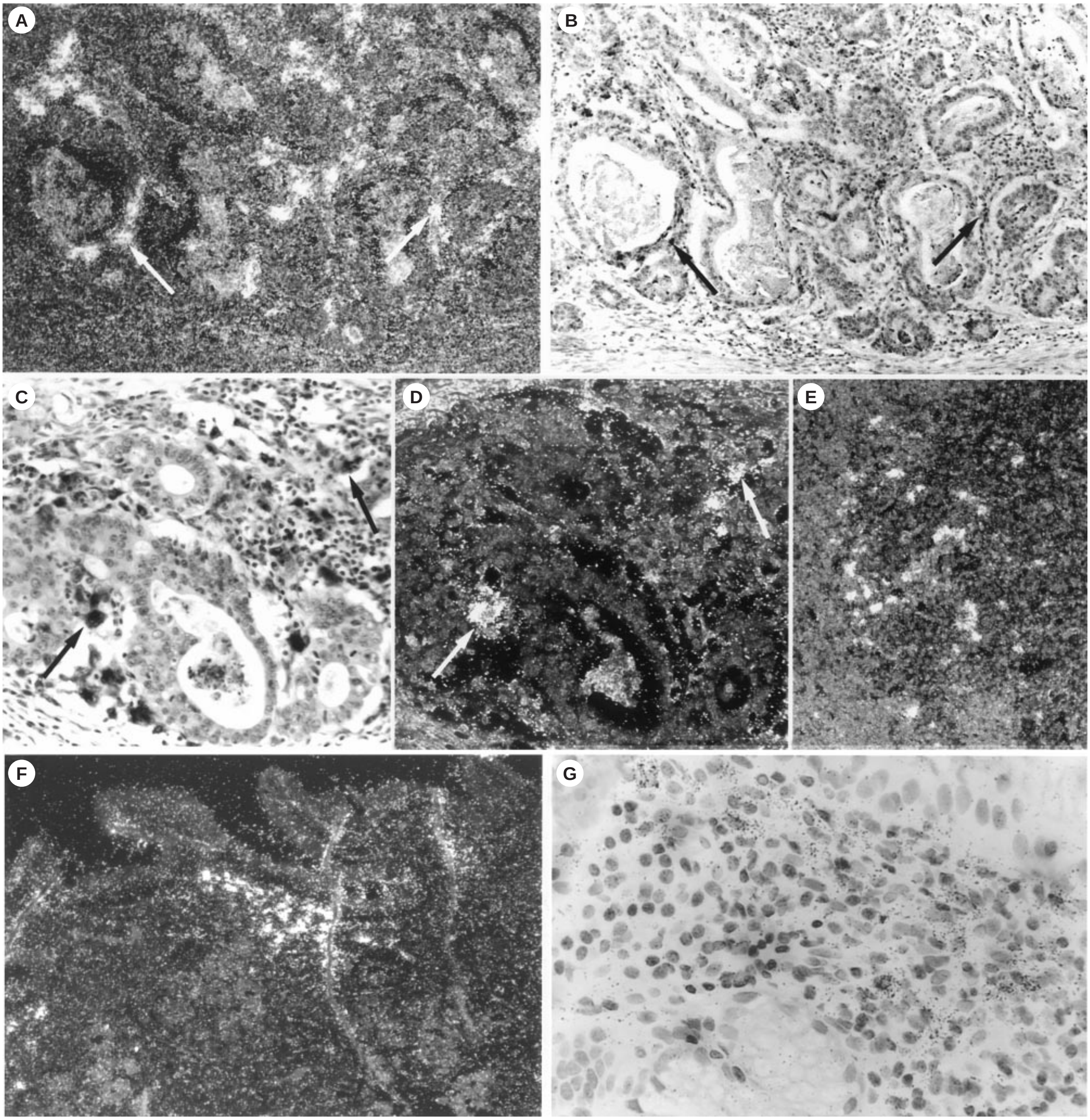

**Figure 3** MMP-12 is expressed by macrophages. (**A**) MMP-12 expression in a well-differentiated area of the tumour shown in Figure 1. (**B**) Corresponding bright-field image. (**C**) MMP-12 is expressed by a subset of macrophages as assessed with immunostaining for macrophage marker CD68 (**D**). Arrows depict corresponding spots. (**E**) Lymph node metastasis with MMP-12 expressing cells surrounding metastatic glands. (**F**) MMP-12 expression in oesophageal mucosa exhibiting intestinal metaplasia. (**G**) High magnification on cells expressing MMP-12 beneath the metaplastic epithelium. Magnification x40 (**E**), x100 (**A, B, F**), x200 (**C, D**), and x400 (**G**).

expressed in areas positive for tenascin. Therefore tenascin-C could well be degraded by matrilysin during invasion and cell migration processes. At least in early breast cancer, tenascin-C expression in the invasion border is a strong predictor of distant metastasis (Jahkola et al, 1996). In addition to matrilysin, also tenascin-C might be a useful marker when progression of BE to adenocarcinoma is evaluated.

We detected stromelysin-2 expression only in few tumours with positive cells located at tumour surface and not in the invasive front. Upregulation of stromelysin-2 has not been detected in gastric, colon and breast adenocarcinomas (McDonnell et al, 1991; Heppner et al, 1996), but it is overexpressed in SCCs of the head and neck (Kerkela et al, 2001). Our results support the hypothesis, that stromelysin-2 expression is limited to epithelial SCCs and not associated with malignant transformation of adenocarcinomas. Stromelysin-2 expression in our samples is probably related to the inflammatory reaction and wound healing-type processes at the luminal surface of tumours.

Surprisingly, signal for collagenase-3 was detected only occasionally in oesophageal adenocarcinomas, although it seems to be

*© 2001 Cancer Research Campaign*

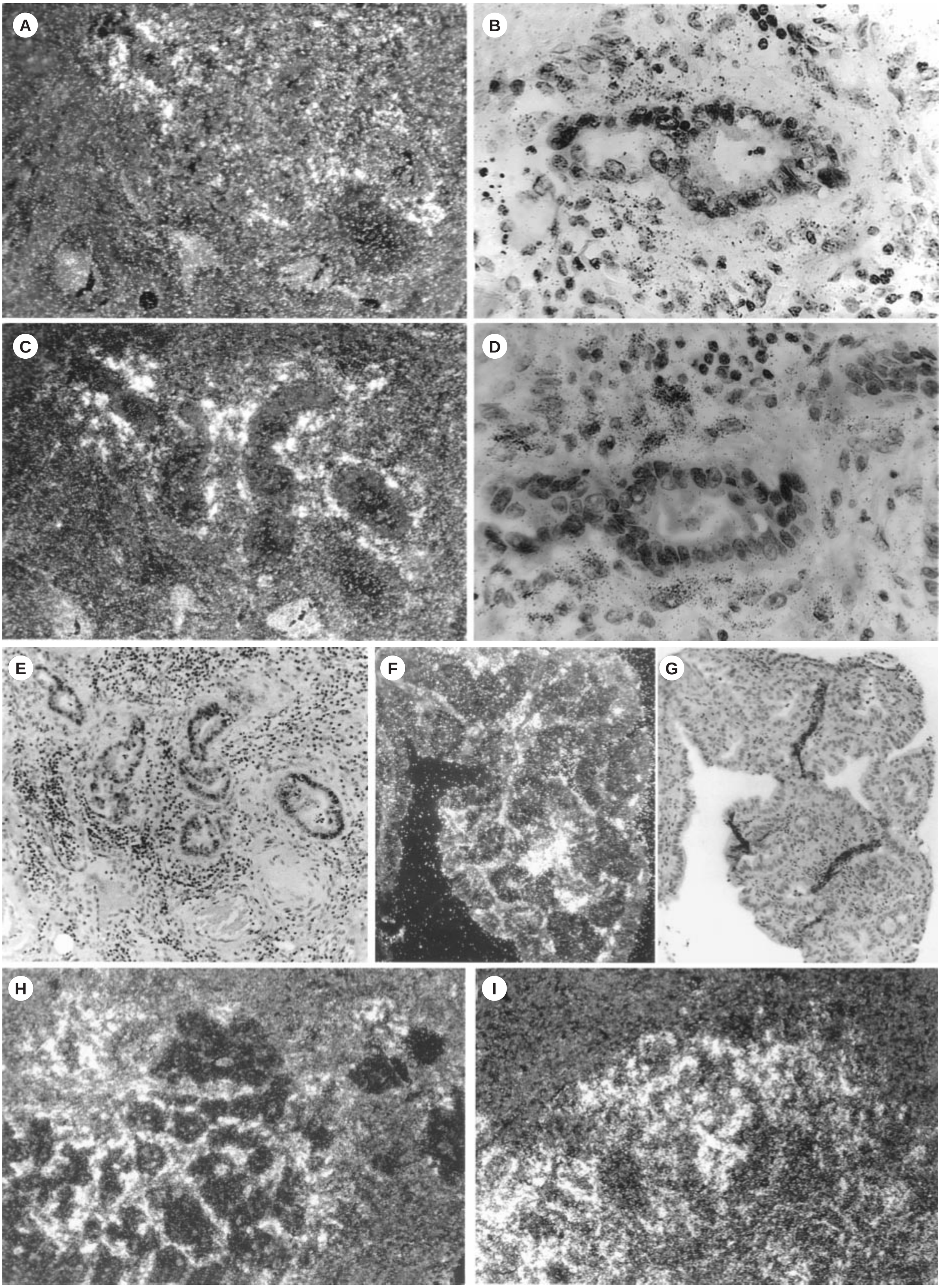

**Figure 4** Expression of TIMPs-1 and -3 by stromal cells in Barrett's carcinoma. (**A**) TIMP-1 expression by activated fibroblast/macrophage-like cells in well-differentiated tumour. (**B**) High magnification on TIMP-1 positive cells. (**C**) TIMP-3 expression in serial section. (**D**) High magnification on TIMP-3 positive activated fibroblast/macrophage-like cells. (**E**) Bright-field image corresponding to picture **C**. (**F**) TIMP-3 is expressed by stromal cells also in intestinal metaplasia. (**G**) Corresponding bright-field image. (**H**) TIMP-1 is abundantly expressed in a metastatic lymph node. (**I**) Also TIMP-3 is expressed in a great number of cells in a metastastatic lymph node. Magnification x100 (**A, C, E–I**), and x400 (**B, D**).

*© 2001 Cancer Research Campaign*

specifically upregulated in SCCs of the head and neck (Johansson et al, 1997), larynx (Cazorla et al, 1998) and vulva (Johansson et al, 1999). While this study was in progress, Etoh et al (2000) demonstrated that in oesophageal SCCs production of MMP-13 is implicated in tumour aggressiveness and prognosis. Thus, collagenase-3 could be a specific marker for transformation of squamous epithelium. In contrast to low production of collagenase-3, we detected collagenase-1 expression in 9/15 tumours and its production was associated with poor differentiation. Cells positive for MMP-1 mRNA were malignant as well as stromal cells. As most MMPs, collagenase-1 is expressed only by stromal cells in colonic and gastric carcinomas, but in oesophageal SCCs also malignant cells produce MMP-1 (Murray et al, 1998).

MMP-12 was expressed by a subset of macrophages as assessed by immunostaining for the macrophage marker CD68. Interestingly, it was more abundantly expressed in well than poorly differentiated tumours. MMP-12 cleaves, at least in vitro, plasminogen to angiostatin which induces apoptosis in endothelial cells (Lucas et al, 1998) and may in this way inhibit angiogenesis. Expression of MMP-12 could reflect host-response to tumour tissue. MMP-12 is induced in monocyte/macrophages by macrophage contact with T cells. Furthermore, several cytokines upregulated in cancers, such as IL-1β, TNF-α, VEGF and GM-CSF, can upregulate MMP-12 secretion by peripheral-blood-derived macrophages (Feinberg et al, 2000).

In addition to MMP-12, also TIMP-1 and -3 have antiangiogenic properties. Like in well-differentiated cutaneous and oral SCCs (Sutinen et al, 1998; Airola et al, 1999), both of these MMP inhibitors were abundantly expressed by stromal fibroblast-like cells. In contrast to TIMP-1, which was expressed in deeper areas of lesions, TIMP-3 was expressed throughout the cancer tissue, and in larger numbers of cells. These observations might reflect different biological roles of these two TIMPs; TIMP-1 is up-regulated after malignant cells protrude into muscle layer and deeper parts of mucosa, whereas TIMP-3 is up-regulated earlier in oncogenesis. The present results further substantiate previous data on a positive correlation between elevated TIMP levels and higher invasiveness (Airola et al, 1999).

In conclusion, our results provide evidence that matrilysin is the principal MMP expressed by malignant cells in Barrett's adenocarcinoma. Furthermore, in our material 4/6 lymph-node metastases and the primary tumours from which they were originated showed matrilysin expression, strongly suggesting a crucial role for matrilysin in invasion and metastasis of Barrett's adenocarcinoma. We also concluded that matrilysin and tenascin-C might be useful markers for evaluating progression of BE to adenocarcinoma.

### ACKNOWLEDGEMENTS

The authors acknowledge Mrs Alli Tallqvist and Mrs Birgitta Arteva for their skillful technical assistance and Drs Lynn Matrisian, Steven Shapiro and Veli-Matti Kähäri for plasmids. This study was supported by grants from the Academy of Finland, the Sigrid Juselius Foundation, Finska Läkaresällskapet and Helsinki University Central Hospital Research Foundation.

### REFERENCES

Adachi Y, Yamamoto H, Itoh F, Hinoda Y, Okada Y and Imai K (1999) Contribution of matrilysin (MMP-7) to the metastatic pathway of human colorectal cancers. *Gut* **45**: 252–258

Ahonen M, Baker AH and Kahari VM (1998) Adenovirus-mediated gene delivery of tissue inhibitor of metalloproteinases-3 inhibits invasion and induces apoptosis in melanoma cells. *Cancer Res* **58**: 2310–2315

Airola K, Ahonen M, Johansson N, Heikkilä P, Kere J, Kähäri V-M and Saarialho-Kere UK (1998) Human TIMP-3 is expressed during fetal development, hair growth cycle and cancer progression. *J Histochem Cytochem* **46**: 437–448

Airola K, Karonen T, Vaalamo M, Lehti K, Lohi J, Kariniemi AL, Keski-Oja J and Saarialho-Kere UK (1999) Expression of collagenases-1 and -3 and their inhibitors TIMP-1 and -3 correlates with the level of invasion in malignant melanomas. *Br J Cancer* **80**: 733–743

Anand-Apte B, Pepper MS, Voest E, Montesano R, Olsen B, Murphy G, Apte SS and Zetter B (1997) Inhibition of angiogenesis by tissue inhibitor of metalloproteinase-3. *Invest Ophthalmol Vis Sci* **38**: 817–823

Antonioli DA and Wang HH (1997) Morphology of Barrett's esophagus and Barrett's-associated dysplasia and adenocarcinoma. *Gastroenterol Clin North Am* **26**: 495–506

Apte SS, Olsen BR and Murphy G (1996) The gene structure of tissue inhibitor of metalloproteinases (TIMP)-3 and its inhibitory activities define the distinct TIMP gene family. *J Biol Chem* **270**: 14313–14318

Bonelli L (1993) Barrett's esophagus: results of a multicentric survey. G.O.S.P.E. (Gruppo Operativo per lo Studio delle Precancerosi Esofagee). *Endoscopy* **25**: 652–654

Brabletz T, Jung A, Dag S, Hlubek F and Kirchner T (1999) Beta-catenin regulates the expression of the matrix metalloproteinase-7 in human colorectal cancer. *Am J Pathol* **155**: 1033–1038

Cazorla M, Hernandez L, Nadal A, Balbin M, Lopez JM, Vizoso F, Fernandez PL, Iwata K, Cardesa A, Lopez-Otin C and Campo E (1998) Collagenase-3 expression is associated with advanced local invasion in human squamous cell carcinomas of the larynx. *J Pathol* **186**: 144–150

Chambers AF and Matrisian LM (1997) Changing views of the role of matrix metalloproteinases in metastasis. *J Natl Cancer Inst* **89**: 1260–1270

Chandler S, Cossins J, Lury J and Wells G (1996) Macrophage metalloelastase degrades matrix and myelin proteins and processes a tumour necrosis factor-alpha fusion protein. *Biochem Biophys Res Commun* **228**: 421–429

Etoh T, Inoue H, Yoshikawa Y, Barnard GF, Kitano S and Mori M (2000) Increased expression of collagenase-3 (MMP-13) and MT1-MMP in oesophageal cancer is related to cancer aggressiveness. *Gut* **47**: 50–56

Feinberg MW, Jain MK, Werner F, Sibinga NE, Wiesel P, Wang H, Topper JN, Perrella MA and Lee ME (2000) Transforming growth factor-beta 1 inhibits cytokine-mediated induction of human metalloelastase in macrophages. *J Biol Chem* **275**: 25766–25773

Fingleton BM, Heppner Goss KJ, Crawford HC and Matrisian LM (1999) Matrilysin in early stage intestinal tumorigenesis. *APMIS* **107**: 102–110

Gronski TJ Jr, Martin RL, Kobayashi DK, Walsh BC, Holman MC, Huber M, Van Wart HE and Shapiro SD (1997) Hydrolysis of a broad spectrum of extracellular matrix proteins by human macrophage elastase. *J Biol Chem* **272**: 12189–12194

Heppner KJ, Lynn MM, Jensen RA and Rodgers WH (1996) Expression of most matrix metalloproteinase family members in breast cancer represents a tumor-induced host response. *Am J Pathol* **149**: 273–282

Jahkola T, Toivonen T, von Smitten K, Blomqvist C and Virtanen I (1996) Expression of tenascin in invasion border of early breast cancer correlates with higher risk of distant metastasis. *Int J Cancer* **69**: 445–447

Jankowski JA, Wright NA, Meltzer SJ, Triadafilopoulos G, Geboes K, Casson AG, Kerr D and Young LS (1999) Molecular evolution of the metaplasia-dysplasia-adenocarcinoma sequence in the esophagus. *Am J Pathol* **154**: 965–973

Johansson N, Airola K, Grenman R, Kariniemi AL, Saarialho-Kere U and Kahari VM (1997) Expression of collagenase-3 (matrix metalloproteinase-13) in squamous cell carcinomas of the head and neck. *Am J Pathol* **151**: 499–508

Johansson N, Vaalamo M, Grenman S, Hietanen S, Klemi P, Saarialho-Kere U and Kahari VM (1999) Collagenase-3 (MMP-13) is expressed by tumor cells in invasive vulvar squamous cell carcinomas. *Am J Pathol* **154**: 469–480

Kahari VM and Saarialho-Kere U (1999) Matrix metalloproteinases and their inhibitors in tumour growth and invasion. *Ann Med* **31**: 34–45

Kerkela E, Ala-aho R, Jeskanen L, Lohi J, Grenman R, Kahari VM and Saarialho-Kere U (2001) Differential patterns of stromelysin-2 (MMP-10) and MT1-MMP (MMP-14) expression in epithelial skin cancers. *Br J Cancer* **84**: 659–669

Koshikawa N, Giannelli G, Cirulli V, Miyazaki K and Quaranta V (2000) Role of cell surface metalloprotease MT1-MMP in epithelial cell migration over laminin-5. *J Cell Biol* **148**: 615–624

Kusukawa J, Harada H, Shima I, Sasaguri Y, Kameyama T and Morimatsu M (1996) The significance of epidermal growth factor receptor and matrix

© 2001 Cancer Research Campaign metalloproteinase-3 in squamous cell carcinoma of the oral cavity. *Eur J Cancer B Oral Oncol* **32B**: 217–221

Lucas R, Holmgren L, Garcia I, Jimenez B, Mandriota SJ, Borlat F, Sim BK, Wu Z, Grau GE, Shing Y, Soff GA, Bouck N and Pepper MS (1998) Multiple forms of angiostatin induce apoptosis in endothelial cells. *Blood* **92**: 4730–4741

Martin DC, Ruther U, Sanchez-Sweatman OH, Orr FW, Khokha R (1996) Inhibition of SV40 T antigen-induced hepatocellular carcinoma in TIMP-1 transgenic mice. *Oncogene* **13**: 569–576

McDonnell S, Navre M, Coffey RJ and Matrisian LM (1991) Expression and localization of the matrix metalloproteinase Pump-1 (MMP-7) in human gastric and colon carcinomas. *Mol Carcinog* **4**: 527–533

Mueller J, Werner M and Siewert JR (2000) Malignant progression in Barrett's esophagus: pathology and molecular biology. *Recent Results Cancer Res* **155**: 29–41

Murphy G, Cockett MI, Ward RV, Docherty AJ (1991) Matrix metalloproteinase degradation of elastin, type IV collagen and proteoglycan. A quantitative comparison of the activities of 95 kDa and 72 kDa gelatinases, stromelysins-1 and -2 and punctuated metalloproteinase (PUMP). *Biochem J* **277**: 277–279

Murray GI, Duncan ME, O'Neil P, McKay JA, Melvin WT and Fothergill JE (1998) Matrix metalloproteinase-1 is associated with poor prognosis in oesophageal cancer. *J Pathol* **185**: 256–261

Nagase H and Woessner JF Jr (1999) Matrix metalloproteinases. *J Biol Chem* **274**: 21491–21494

Ohashi K, Nemoto T, Nakamura K and Nemori R (2000) Increased expression of matrix metalloproteinase 7 and 9 and membrane type 1-matrix metalloproteinase in esophageal squamous cell carcinomas. *Cancer* **88**: 2201–2209

Park HI, NI J, Gerkema FE, Liu D, Belozerov VE and Sang QX (2000) Identification and characterization of human endometase (Matrix metalloproteinase-26) from endometrial tumor. *J Biol Chem* **275**: 20540–20544

Peters JH, Clark GW, Ireland AP, Chandrasoma P, Smyrk TC and DeMeester TR (1994) Outcome of adenocarcinoma arising in Barrett's esophagus in endoscopically surveyed and nonsurveyed patients. *J Thorac Cardiovasc Surg* **108**: 813–821

Pyke C, Romer J, Kallunki P, Lund LR, Ralfkiaer E, Dano K and Tryggvason K (1994) The γ2-chain of kalinin/laminin-5 is preferentially expressed in invading malignant cells in human cancers. *Am J Pathol* **145**: 782–791

Pyke C, Salo S, Ralfkiaer E, Romer J, Dano K and Tryggvason K (1995) Laminin-5 is a marker of invading cancer cells in some human carcinomas and is coexpressed with the receptor for urokinase plasminogen activator in budding cancer cells in colon adenocarcinomas. *Cancer Res* **55**: 4132–4139

Saarialho-Kere U, Chang ES, Welgus HG and Parks WC (1992) Distinct localization of collagenase and TIMP expression in wound healing associated with ulcerative pyogenic granuloma. *J Clin Invest* **90**: 1952–1957

Saarialho-Kere UK, Pentland AP, Birkedal-Hansen H, Parks WC and Welgus HG (1994) Distinct populations of basal keratinocytes express stromelysin-1 and stromelysin-2 in chronic wounds. *J Clin Invest* **94**: 79–88

Saarialho-Kere U, Vaalamo M, Puolakkainen P, Airola K, Parks WC and Karjalainen-Lindsberg M-L (1996) Enhanced expression of matrilysin, collagenase and stromelysin-1 in gastrointestinal ulcers. *Am J Pathol* **148**: 519–526

Shima I, Sasaguri Y, Kusukawa J, Yamana H, Fujita H, Kakegawa T and Morimatsu M (1992) Production of matrix metalloproteinase-2 and metalloproteinase-3 related to malignant behavior of esophageal carcinoma. A clinicopathologic study. *Cancer* **70**: 2747–2753

Siri A, Knauper V, Veirana N, Caocci F, Murphy G and Zardi L (1995) Different susceptibility of small and large human tenascin-C isoforms to degradation by matrix metalloproteinases. *J Biol Chem* **270**: 8650–8654

Skyldberg B, Salo S, Eriksson E, Aspenblad U, Moberger B, Tryggvason K and Auer G (1991) Laminin-5 as a marker of invasiveness in cervical lesions. *J Natl Cancer Inst* **91**: 1882–1887

Sutinen M, Kainulainen T, Hurskainen T, Vesterlund E, Alexander JP, Overall CM, Sorsa T and Salo T (1998) Expression of matrix metalloproteinases (MMP-1 and -2) and their inhibitors (TIMP-1, -2 and -3) in oral lichen planus, dysplasia, squamous cell carcinoma and lymph node metastasis. *Br J Cancer* **77**: 2239–2245

Tanaka M, Itai T, Adachi M and Nagata S (1998) Downregulation of Fas ligand by shedding. *Nat Med* **4**: 31–36

Tani T, Lumme A, Linnala A, Kivilaakso E, Kiviluoto T, Burgeson RE, Kangas L, Leivo I and Virtanen I (1997) Pancreatic carcinomas deposit laminin-5, preferably adhere to laminin-5, and migrate on the newly deposited basement membrane. *Am J Pathol* **151**: 1289–1302

Vaalamo M, Weckroth M, Puolakkainen P, Kere J, Saarinen P, Lauharanta J and Saarialho-Kere UK (1996) Patterns of matrix metalloproteinase and TIMP-1 expression in chronic and normally healing human cutaneous wounds. *Br J Dermatol* **135**: 52–59

Vaalamo M, Mattila L, Johansson N, Kariniemi A-L, Karjalainen-Lindsberg M-L, Kähäri V-M and Saarialho-Kere UK (1997) Distinct populations of stromal cells express collagenase-3 (MMP-13) and collagenase-1 (MMP-1) in chronic ulcers but not in normally healing wounds. *J Invest Dermatol* **109**: 96–101

Vaalamo M, Karjalainen-Lindsberg M-L, Puolakkainen P, Kere J and Saarialho-Kere U (1998) Distinct expression profiles of stromelysin-2 (MMP-10), collagenase-3 (MMP-13), macrophage metalloelastase (MMP-12), and tissue inhibitor of metalloproteinases-3 (TIMP-3) in intestinal ulcerations. *Am J Pathol* **152**: 1005–1014

van Sandick JW, van Lanschot JJ, Kuiken BW, Tytgat GN, Offerhaus GJ and Obertop H (1998) Impact of endoscopic biopsy surveillance of Barrett's oesophagus on pathological stage and clinical outcome of Barrett's carcinoma. *Gut* **43**: 216–222

Velasco G, Pendas AM, Fueyo A, Knauper V, Murphy G and Lopez-Otin C (1999) Cloning and characterization of human MMP-23, a new matrix metalloproteinase predominantly expressed in reproductive tissues and lacking conserved domains in other family members. *J Biol Chem* **274**: 4570–4576

Westermarck J and Kahari VM (1999) Regulation of matrix metalloproteinase expression in tumor invasion. *FASEB J* **13**: 781–792

Yamamoto H, Adachi Y, Itoh F, Iku S, Matsuno K, Kusano M, Arimura Y, Endo T, Hinoda Y, Hosokawa M and Imai K (1999) Association of matrilysin expression with recurrence and poor prognosis in human esophageal squamous cell carcinoma. *Cancer Res* **59**: 3313–3316

Yamashita K, Mori M, Shiraishi T, Shibuta K and Sugimachi K (2000) Clinical significance of matrix metalloproteinase-7 expression in esophageal carcinoma. *Clin Cancer Res* **6**: 1169–1174

 *© 2001 Cancer Research Campaign*
